# Form and function in gene regulatory networks: the structure of network motifs determines fundamental properties of their dynamical state space

**DOI:** 10.1098/rsif.2016.0179

**Published:** 2016-07

**Authors:** S. E. Ahnert, T. M. A. Fink

**Affiliations:** 1 Theory of Condensed Matter Group, Cavendish Laboratory, University of Cambridge, JJ Thomson Avenue, Cambridge CB3 0HE, UK; 2 London Institute of Mathematical Sciences, 35A South Street, London W1K 2XF, UK

**Keywords:** network motif, Boolean network, feed-forward loop, gene regulatory network, basin entropy

## Abstract

Network motifs have been studied extensively over the past decade, and certain motifs, such as the feed-forward loop, play an important role in regulatory networks. Recent studies have used Boolean network motifs to explore the link between form and function in gene regulatory networks and have found that the structure of a motif does not strongly determine its function, if this is defined in terms of the gene expression patterns the motif can produce. Here, we offer a different, higher-level definition of the ‘function’ of a motif, in terms of two fundamental properties of its dynamical state space as a Boolean network. One is the basin entropy, which is a complexity measure of the dynamics of Boolean networks. The other is the diversity of cyclic attractor lengths that a given motif can produce. Using these two measures, we examine all 104 topologically distinct three-node motifs and show that the structural properties of a motif, such as the presence of feedback loops and feed-forward loops, predict fundamental characteristics of its dynamical state space, which in turn determine aspects of its functional versatility. We also show that these higher-level properties have a direct bearing on real regulatory networks, as both basin entropy and cycle length diversity show a close correspondence with the prevalence, in neural and genetic regulatory networks, of the 13 connected motifs without self-interactions that have been studied extensively in the literature.

## 1. Introduction

Network motifs, which are small subgraphs of directed networks, have been the subject of much research over the past decade [1,2], and certain motifs, such as the feed-forward loop, are known to play an important role in gene regulatory networks [3]. It has been shown that some motifs appear much more frequently than others in real-world networks, and that the signatures of their relative enrichments with respect to a null model are similar for wide classes of networks. Motifs can thus be used to classify networks into ‘superfamilies’ [2]. In a parallel development, Boolean networks, which were originally studied in the context of large random networks [4,5], have emerged as models of specific gene regulatory networks, particularly in the context of plant development [6,7] and the cell cycle [8,9]. While the relative prevalences of network motifs can be understood as a topological, static property of a network, Boolean networks offer a way to measure the dynamical capability of a network. In this work, we study the Boolean dynamics of network motifs in order to explore the relationship between structure and dynamics, and, more broadly, between form and function. In biology, the form of the phenotype is often strongly determined by its function. This is true at the level of organisms, which exhibit a remarkable degree of specialization according to the characteristics of their environment, such as their predators (e.g. in the form of camouflage) and prey (e.g. the anteater's snout). It also holds true down to the molecular level, at which proteins evolve in order to form protein complexes of very specific structures [10,11]. It seems reasonable that a link between form and function should also hold in biological networks, which is why the existence of such links has been investigated in the recent literature [12–14], including through Boolean networks of network motifs [15]. The results of this work have suggested that the form and function of network motifs are not closely linked because: (i) the structure of network motifs does not strongly determine its functional versatility, if this is defined in terms of initial and final states and (ii) a given functional task (again defined in terms of initial and final states) can be performed by more than one structure. Earlier work in this direction, employing other models, has similarly found that the structure of a motif does not determine its function, if ‘function’ is defined in terms of the variety of gene expression patterns that can be produced by the motif [12–14].

In this work, we examine the link between the form and function of network motifs differently, by defining their functional versatility in terms of two structural properties of their dynamical attraction basins. The first property is basin entropy, which serves as a measure of dynamical complexity in Boolean networks [16,17]. The second is the number of different attractor cycle lengths that can be realized in state space for a given network motif. We show that both of these measures strongly depend on the presence of feedback loops and feed-forward loops in the motif. These two loops are the simplest triangle motifs and have been shown to play an important role in biological regulatory networks [2,3,18]. In particular, we demonstrate that motifs containing a three-node feedback loop have increased basin entropy and a larger diversity of attractor cycle lengths. By contrast, motifs that contain a feed-forward loop and no three-node feedback loop have particularly low basin entropy and low cycle length diversity. Finally, we connect these results to the frequencies of motifs in real-world gene regulatory networks and signal-transduction networks [2]. We show that both basin entropy and cycle length diversity are inversely correlated with the relative enrichment of network motifs, meaning that motifs containing feedback loops are suppressed in real-world regulatory networks. In summary, the structural properties of network motifs strongly determine their functional versatility as regulatory circuits, if we define this functional versatility in terms of attractor properties.

## 2. Boolean dynamics of network motifs

**Figure 1.**
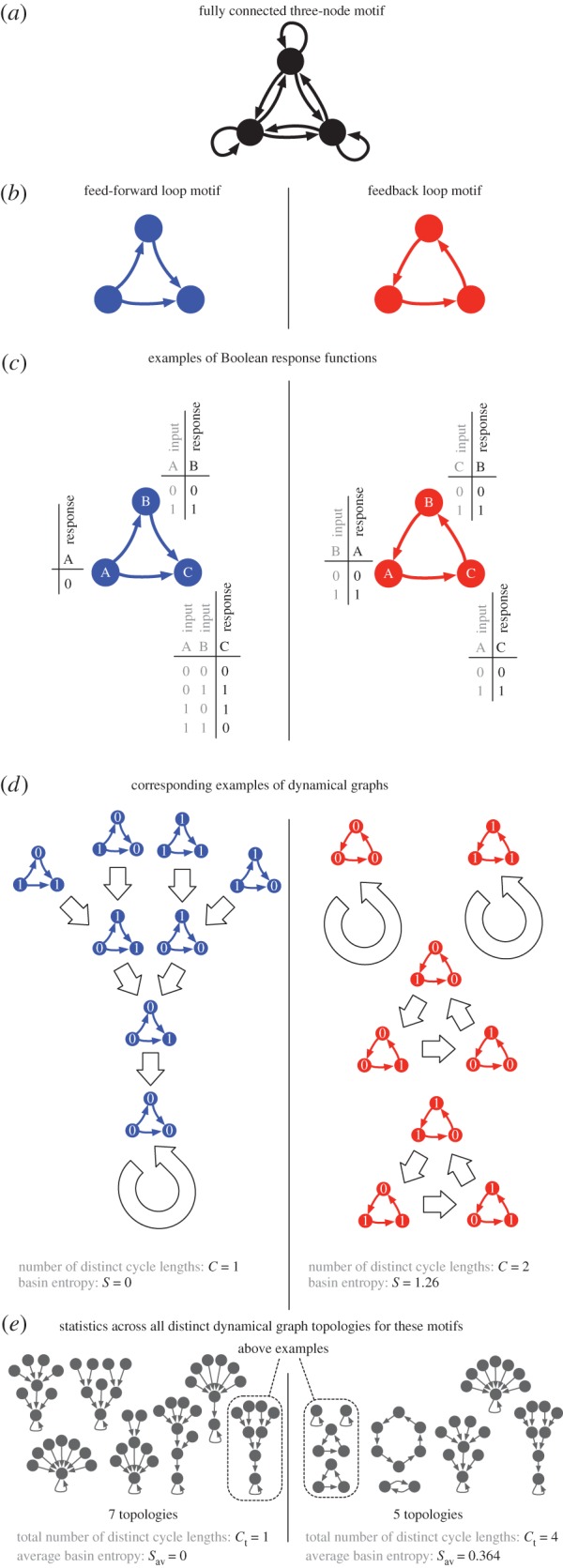
Illustration of motifs as Boolean networks. (*a*) The fully connected three-node motif, showing all nine possible connections. (*b*) Two example motifs, the feed-forward loop (left) and the feedback loop (right). (*c*) Examples of Boolean functions that could be placed on these example motifs. The binary node states are updated simultaneously according to the table of update rules for each node, and the inputs to that node. (*d*) The dynamical graphs for these example Boolean functions, showing the resulting transitions (shown as broad arrows) between the eight possible states of these three-node motifs. (*e*) All of the different topologies of dynamical graphs that can occur for these two motifs, with the example topologies of step (*d*) highlighted, and two measurements of these dynamical graph topologies: the average basin entropy *S*_av_ and the total number of different cycle lengths *C*_t_.

The fully connected three-node network motif with all nine possible edges is depicted in figure 1*a*, while figure 1*b* shows examples of two simpler three-node network motifs with just three edges: a feed-forward loop and a three-node feedback loop. Similar to Payne & Wagner [15], we study the dynamics of such three-node network motifs using Boolean dynamics. A Boolean network is a directed network in which each node has a binary state (0 or 1), and each node with *k* inputs is associated with a string of 2*^k^* bits. This string, a so-called Boolean function, is an update rule. In each time step, all nodes are updated according to their Boolean function, which specifies the state of the node for any of the 2*^k^* possible combinations of the *k* inputs from other node states (figure 1*c*). In a Boolean network of *N* nodes, the dynamics determined by the Boolean functions gives rise to a directed transition graph of 2*^N^* nodes, representing all the 2*^N^* possible states of the network. We will refer to such graphs as a *dynamical graph*, and each motif can have several of these, corresponding to different Boolean functions. If we ignore the specific combinations of states that the nodes in the dynamical graph correspond to, and only focus on their connectivity, then many dynamical graphs become isomorphic, meaning that they share the same *dynamical graph topology*.

### 2.1. Characterizing the state space of network motifs

The nine possible edges of the three-node motif (shown in figure 1*a*) give rise to 2^9^ = 512 possible ways of connecting three nodes. Many of these however are equivalent (such as three rotated versions of the feed-forward loop shown in figure 1*b*, left). If one takes into account all such equivalencies, 104 topologically distinct three-node motifs remain. By calculating all dynamical transition graphs (such as those shown in figure 1*d*) and comparing their unlabelled topologies one arrives at 951 distinct dynamical graph topologies (such as those shown in figure 1*e*). In contrast with previous work [15], which used pairs of specific initial and final network states to measure the functionality of motifs, our approach is to use dynamical graph topologies. The two dynamical graphs that arise from the two motifs in figure 1*b* with the Boolean functions shown in figure 1*c* are shown in figure 1*d*. Because the three nodes of the feed-forward loop have two, one and zero inputs, respectively, there are 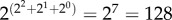 possible configurations of Boolean functions in this motif, one of which is shown in figure 1*c* (left). The 128 configurations for the feed-forward loop can lead to seven different dynamical graph topologies (figure 1*e*, left). For the feedback loop (in which each node has one input), there are 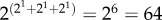 possible configurations, one of which is shown in figure 1*c* (right). These 64 configurations give rise to altogether five different dynamical graph topologies (figure 1*e*, right). We can characterize each dynamical graph topology in terms of two properties. The first is the basin entropy, which was introduced as a complexity measure for Boolean networks in [16,17]. It is defined as
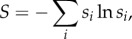
where *s_i_* is the size of the *i*th attraction basin as a fraction of the total state space. For the feed-forward loop with the example of Boolean functions in figure 1*c* (left), the dynamical graph (shown in figure 1*d*, left) only has one attraction basin, so *s*_1_ = 1 and *S* = 0. For the three-node feedback loop example (figure 1*d*, right), we have two basins of size 1 and two of size 3, so that *s*_1_ = 1/8, *s*_2_ = 1/8, *s*_3_ = 3/8 and *s*_4_ = 3/8, giving a basin entropy of *S* = 1/4 ln (1/8) + 3/4 ln (3/8) = ln 8 − (3/4) ln 3 ≈ 1.26. The basin entropy is minimal when all states lie in one attraction basin and maximal when all states form separate attractors.

The second measure we can use to characterize a dynamical graph topology is the number of distinct cycle lengths *C* in the dynamical graph. In the feed-forward dynamical graph example of figure 1*d* (left), we only have one cycle of length 1, i.e. *C* = 1, because we reach a fixed point when all three nodes have state 0. The feedback example (figure 1*d*, right) on the other hand has two cycles of length 1 and two cycles of length 3, and therefore two distinct cycle lengths, or *C* = 2.

For both of these measures, *S* and *C*, we can now define aggregated versions for each motif: the average basin entropy *S*_av_ is calculated across all distinct dynamical graph topologies for a given motif, which are found by considering all possible Boolean functions for that motif. Similarly, the total number of distinct cycle lengths *C*_t_ is also determined across all of these topologies. The value of *S*_av_ for the seven topologies of the feed-forward loop is zero, as all have a single attraction basin that fills the entire state space, and *C*_t_ = 1, as all of these basins have a 1-cycle as their attractor. For the feedback loop, on the other hand, two of the five topologies consist of more than one basin, resulting in a value of *S*_av_ = 0.364. We have *C*_t_ = 4 for the feedback loop, as we find four different cycle lengths (1, 2, 3 and 6) across the five dynamical graph topologies.

### 2.2. Characterizing the structure of network motifs

**Figure 2.**
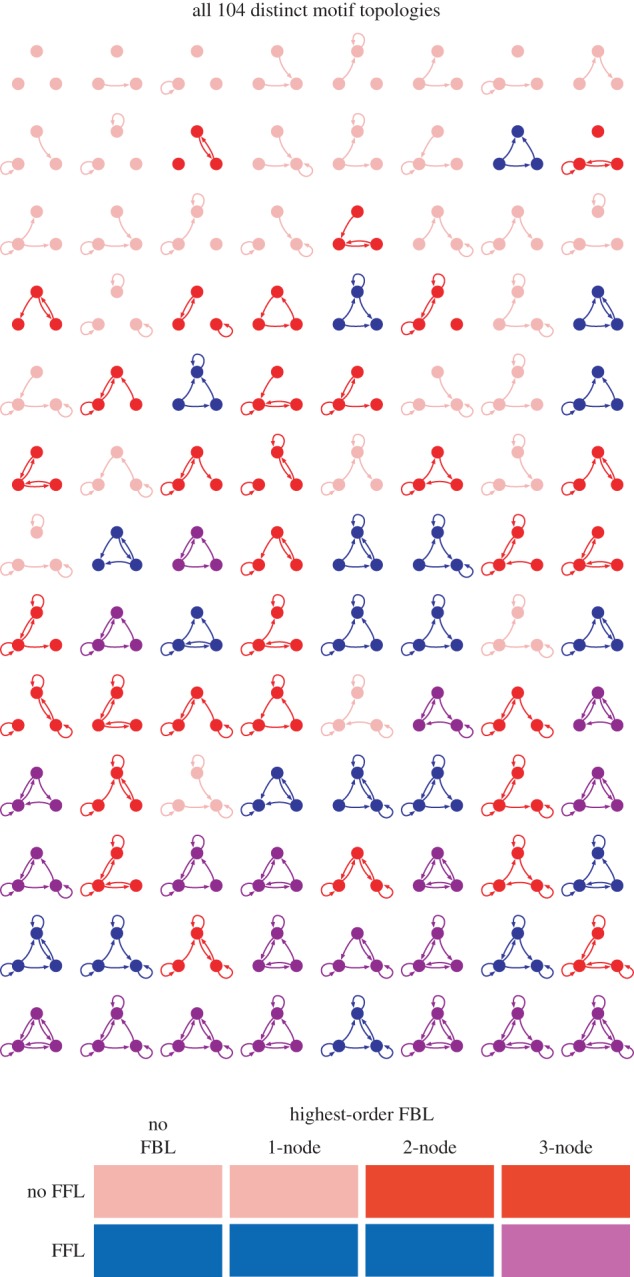
All 104 topologically distinct motifs, colour-coded according to the four categories described in the text. These are motifs containing (i) no feed-forward loops, and no two-node or three-node feedback loops (pink), (ii) no feed-forward loops, and two-node or three-node feedback loops (red), (iii) feed-forward loops and no three-node feedback loops (blue), and (iv) feed-forward loops and three-node feedback loops (purple).

Our overall aim is to establish whether the structure of a motif determines its dynamical behaviour, and thus its function as a regulatory unit. So far, we have introduced two quantities, *S*_av_ and *C*_t_, which allow us to characterize the dynamical state space of a motif. For an effective comparison of structure and dynamics, we also need to characterize the structure of the motifs. We do this by describing the 104 distinct motifs in terms of feed-forward loops and feedback loops. We already used both the feed-forward loop and the three-node feedback loop as examples in figure 1. These two motifs are the two simplest triangle motifs, and can therefore be viewed as basic units of any three-node triangle motif, which is also why other network measures have been defined using them as a basis [19,20]. The feed-forward loop is known to play an important role in biological regulatory networks [2,3], and feedback loops occur for example in the context of negative self-regulatory circuits [18]. For our classification of motif structure, we also consider two-node and one-node feedback loops (also referred to as self-loops or self-interactions). We partition the 104 motifs into four classes of roughly equal size: (i) motifs that contain no feed-forward loops and no two-node or three-node feedback loops, (ii) motifs that contain no feed-forward loops, but do contain two-node or three-node feedback loops, (iii) motifs that contain feed-forward loops, but no three-node feedback loops, and (iv) motifs that contain feed-forward loops and three-node feedback loops. These four categories represent the four possible combinations of the presence or absence of the two basic loops in network motifs, the feedback loop and the feed-forward loop. Figure 2 shows all 104 motifs colour-coded according to this classification.

**Figure 3.**
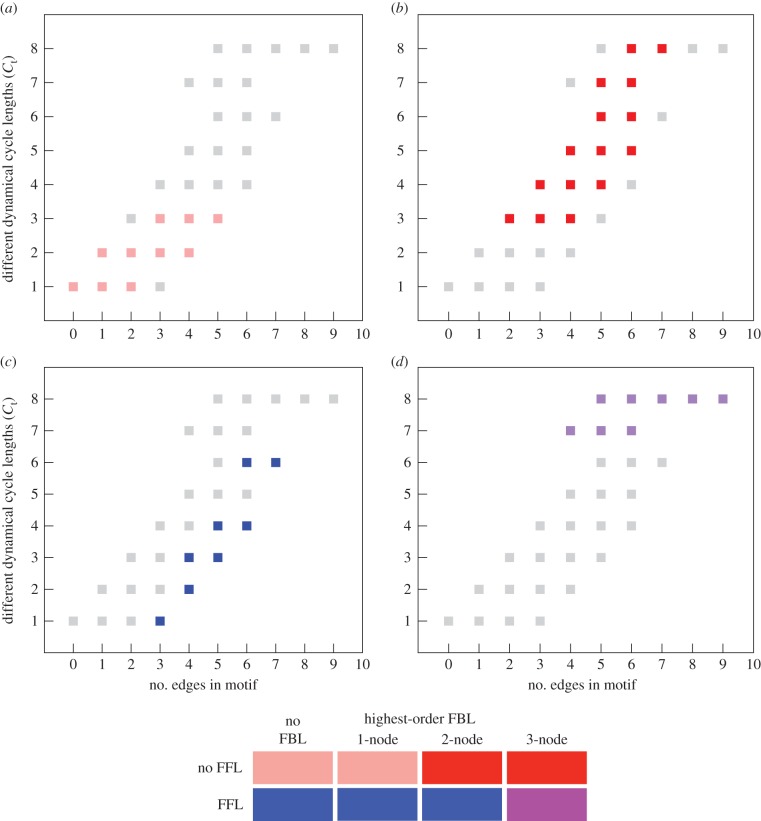
(*a*–*d*) The total number of different cycle lengths *C*_t_ that can be realized with motifs of a given number of edges. All four panels show the values for all 104 motifs, but with different categories highlighted. These are the four structural classes of motifs defined in the text. The colour code is given below the four panels. Motifs with feed-forward loops and without three-node feedback loops (blue) exhibit few cycle lengths for a given number of edges, while those motifs without feed-forward loops and with two-node and three-node feedback loops (red) show high numbers of different cycle lengths. Motifs with only 1-node feedback loops or no loops at all show few cycles (pink), while those with feed-forward and three-node feedback loops can accommodate many cycle lengths (purple).

Figure 3 shows the total number of distinct cycle lengths *C*_t_ for the 104 motifs as a function of the number of edges in the motifs and broken down into the four structural categories described above. As the number of edges in a Boolean network is closely related to the total length of the Boolean functions, it represents a proxy for the potential processing power of the network. Accordingly, the values of *C*_t_ for all 104 motifs show a broad positive correlation with the number of edges. For a given number of edges, however, there are clear differences in the values of *C*_t_ for the motifs in our four categories. Motifs with a feed-forward loop but without a three-node feedback loop show the least cycle diversity of *C*_t_ for a given number of edges (shown in figure 3*c* in blue). Motifs with no feed-forward loop and two-node or three-node feedback loops on the other hand exhibit high values of *C*_t_ for a given number of edges (figure 3*b*, red), meaning that they are capable of producing many different cycle lengths in their dynamical behaviours. Motifs with no feed-forward loops and no two-node or three-node feedback loops have universally low *C*_t_ values (figure 3*a*, pink), while motifs with feed-forward loops and three-node feedback loops have universally high *C*_t_ values (figure 3*d*, purple). Note that there are motifs with four and five edges in both of these extreme categories, which shows how much the dynamical complexity of a motif depends on the arrangement, rather than the number, of its edges.

**Figure 4.**
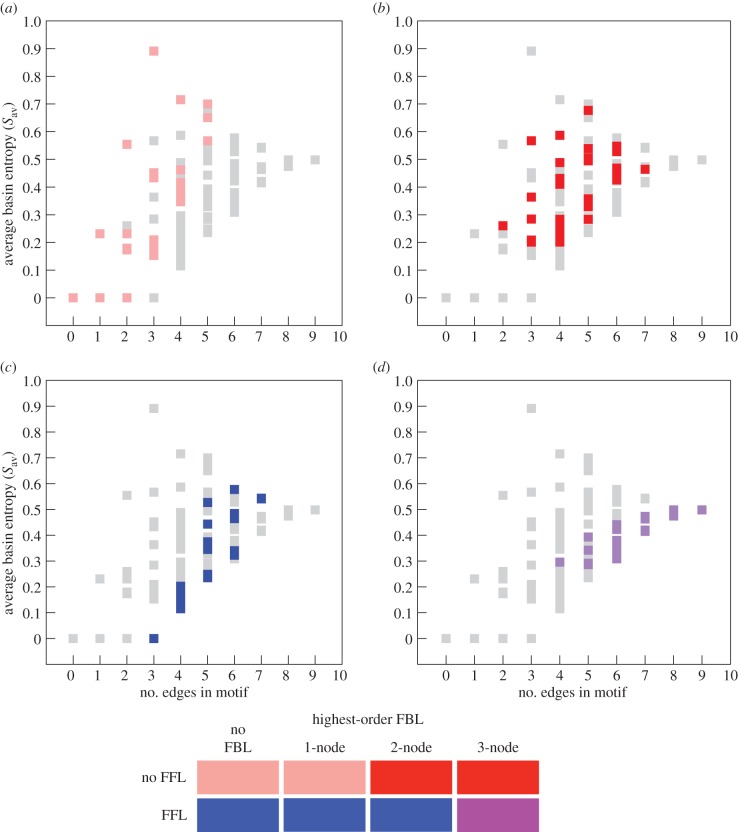
(*a*–*d*) The average basin entropy *S*_av_ measures the fragmentation of the dynamical graphs of a motif. This quantity is shown here against the number of edges in each motif. All four panels show the values for all 104 motifs, but with different categories highlighted. These are the four structural classes of motifs defined in the text. The colour code is given below the four panels. Motifs with feed-forward loops and without three-node feedback loops (blue) exhibit relatively low values of *S*_av_ for a given number of edges, while those motifs without feed-forward loops and with feedback loops (pink and red) exhibit higher values of *S*_av_ for a given number of edges. Note that the three values for *S*_av_ = 0 and 0, 1 and 2 edges correspond to motifs without any loops at all. Motifs with feed-forward and three-node feedback loops (purple) have intermediate values of *S*_av_.

Similarly, figure 4 shows the average basin entropy *S*_av_ of the 104 motifs as a function of the number of edges, for the four structural categories. The largest values of the basin entropy are displayed by motifs with 1-node feedback loops (figure 4*a*, in pink). Note that the five motifs that have no loops of any kind have average basin entropy *S*_av_ = 0. These are in the same structural category as the 1-node feedback loops, and are represented in figure 4 by the three points at *S*_av_ = 0 and 0, 1 and 2 edges. For a given number of edges, motifs that contain a feed-forward loop and no three-node feedback loops tend to display lower *S*_av_ values (figure 4*c*, in blue), and motifs that contain two-node and three-node feedback loops and no feed-forward loop display higher *S*_av_ values (figure 4*b*, red). Motifs that contain both feed-forward loops and three-node feedback loops exhibit relatively low basin entropy for a given number of edges, but the total range of *S*_av_ values narrows considerably with increasing numbers of edges (figure 4*d*, purple). These results show that the average basin entropy offers a complementary definition of dynamical complexity to *C*_t_. Between them these two measures reveal that there is indeed a strong connection between certain structural characteristics of a motif and aspects of its dynamical behaviour. The values of *S*_av_ and *C*_t_ for all 104 motifs can be found in the electronic supplementary material.

### 2.3. Real-world regulatory networks

**Figure 5.**
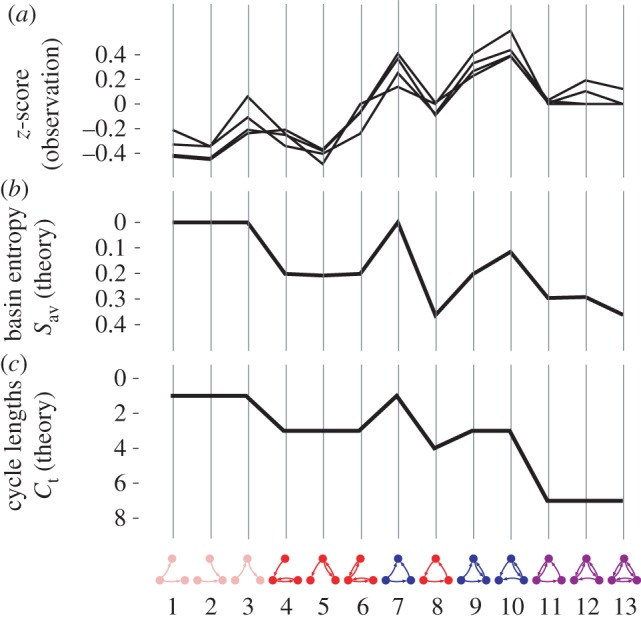
This figure compares the average basin entropy *S*_av_ and the total number of distinct cycles *C*_t_ with the enrichments, in four real-world regulatory and neural networks, of the 13 connected three-node motifs without self-interactions. The four networks are two developmental transcription networks in fruit fly and sea urchin, a signal-transduction network of mammalian cells, and the neural network of *Caenorhabditis elegans*. These data are adapted directly from the seminal study of these 13 motifs in [2] and were kindly made available by the authors of this work. (*a*) The enrichment profiles for these networks are shown relative to a null model, in the form of a *z*-score. Note that the motifs without any feedback loops (7, 9 and 10) are the most prevalent. (*b*) The values of the basin entropy *S*_av_ are shown for the same motifs on an inverted scale, and exhibit a striking similarity to the motif *z*-scores. On this inverted scale, the former shows a slight downwards trend with increasing edge number in the motifs, whereas the latter shows a slight upwards trend. The successive gradients of these two profiles however match almost perfectly. To separate the effects of edge number from other characteristics of motif structure, we compare the successive differences in *z*-score between motifs Δ*Z* with the differences in average basin entropy Δ*S*_av_ and find that these show a strong correlation with a Pearson coefficient of −0.7862. (*c*) The values of *C*_t_, the total number of distinct cycle lengths, for these motifs, which follow a similar pattern to the *z*-scores and *S*_av_. The correlation of the successive differences Δ*Z* and Δ*C*_t_ is similarly high (−0.8166). Note that *C*_t_, like *S*_av_, is shown on an inverted scale.

We now consider a subset of the 104 motifs, namely the 13 connected motifs without self-interactions. This important subclass is of interest as it has been studied extensively in the literature, and was the basis for the seminal work on network motifs, by Alon and co-workers [1,2]. The frequencies of these 13 motifs, relative to a null model, were used to define ‘superfamilies’ of networks that exhibit similar motif frequency distributions. One such superfamily included signal-transduction networks in mammalian cells, neural networks and transcription networks. The enrichment signature across the 13 motifs, expressed in the form of a *z*-score to show the deviation from the null model, is adapted directly from [2] in figure 5*a*. In figure 5*b,c*, we show the values of *S*_av_ and *C*_t_, respectively, for the same motifs, plotted on an inverted scale. The enrichment profiles show a striking similarity to those of *S*_av_ and *C*_t_, which becomes even clearer if we compare the gradients of the series rather than the absolute values. The Pearson correlation between the successive changes in basin entropy and those in the motif enrichments for real-world networks is −0.7862 (*p*-value: 3.54 × 10^−11^), and the equivalent correlation for the number of cycle lengths is −0.8166 (*p*-value: 1.50 × 10^−12^). If we compare the profiles with the structural classification of the motifs, shown underneath them in figure 5, we see that the lower values of the motif enrichment (and, correspondingly, the higher values of the basin entropy and cycle length number, plotted on an inverted scale) occur when the motif contains a three-node feedback loop (motifs 8, 11, 12 and 13 in figure 5) and to a lesser extent when it contains a two-node feedback loop (motifs 4, 5, 6). This indicates that feedback loops are suppressed in these networks relative to the feed-forward loop. The close correlation with the dynamical complexity of Boolean network motifs as measured by the basin entropy suggests that the fragmentation of state space may be a reason why the feedback loop motif appears to be less desirable in real-world regulatory networks. Only those triangle motifs (7, 9 and 10) which do *not* contain any feedback loops are highly enriched. These results also underline the importance of the feed-forward loop, which has been established as an important building block of biological networks [3]. Note that while *S*_av_ and *C*_t_ are strongly correlated (Pearson: 0.8705) for the 13 connected motifs without self-interactions, they are only weakly correlated (Pearson: 0.4092) across all 104 motifs.

## 3. Discussion

In our structural classification of the 104 networks into four categories, we chose to group networks with two-node and three-node feedback loops together if they did not contain feed-forward loops. The reasons for this were that this classification produced roughly equal-sized categories, and that the dynamical behaviours of these networks were sufficiently similar. This did not hold for networks with two-node and three-node feedback loops that did contain feed-forward loops. In these, the networks with only a two-node feedback loop exhibited very different dynamics from those containing a three-node feedback loop, and had more in common with networks that had only a one-node feedback loop or none at all.

Feedback loops have been shown to occur in some regulatory networks, but only in small numbers in transcriptional networks [2,21]. The likely reasons are that feedback loops tend to consist of a transcriptional regulation and a protein–protein interaction, such as the much studied p53-MDM2 loop [18]. One of these regulations is typically a negative one, to enable self-regulation, as purely positive feedback loops would probably lead to an irreversible—and therefore highly undesirable—cellular state [22]. The danger of such runaway states may be another reason why feedback loops are uncommon. Perhaps the most prominent example of a transcriptional feedback loop is an artificial one, the repressilator [23], which exhibits noisy performance, offering another reason why purely transcriptional feedback loops are suppressed.

The values of *S*_av_ and *C*_t_ both show a slight downwards trend (on the inverted scale) with increasing edge number in the motifs, whereas the motif *z*-scores show a slight upwards trend. A likely reason for this relative divergence could be that a larger number of edges in a motif is likely to result in more complex partitions of state spaces, which in turn mean higher basin entropy and greater cycle length diversity. One would therefore expect an underlying trend towards higher basin entropy and cycle length number with increasing edge number. As we show above, we can control for this underlying effect by considering the gradients of the profiles, for which we observe strong correlations between the gradients of the *z*-scores and the gradients of *S*_av_ and *C*_t_.

## 4. Conclusion

A number of publications have addressed the link between the structure and dynamics or, more broadly speaking, form and function of network motifs [12–15]. In this literature, ‘function’ has been defined in terms of gene expression patterns, which has led to the conclusion that a single structure can lead to a wide variety of functional behaviours in this sense. Form therefore does not dictate function on this level. If we however define function on a higher level, in terms of topological properties of the state space, a clear relationship between structure and function emerges. That this relationship is likely to be meaningful is indicated by the close correspondence between the topological properties—basin entropy and cycle length number—and the enrichments of motifs in regulatory networks and neural networks. The presence of feedback loops in a motif results in a profoundly different organization of state space, even if the feedback loops are among feed-forward loops. This is also why the highly stable, unfragmented attraction basins of simpler feed-forward loops provide a reliable template for the implementation of a variety of gene expression patterns (which, in other contexts such as [12–15], would be described as different ‘functions’ of the circuit). It is highly unlikely that these fundamental topological properties of the state space do not influence the biological evolution of regulatory networks.

## Supplementary Material

C_t and S_av for all 104 network motifs
